# The influence of water hardness perturbations on bubble departure dynamics

**DOI:** 10.1038/s41598-021-00375-7

**Published:** 2021-10-25

**Authors:** P. Dzienis, R. Mosdorf, J. Czarnecki

**Affiliations:** Faculty of Mechanical Engineering, Bialystok University of Technology, Wiejska 45C, 15-351 Białystok, Poland

**Keywords:** Mechanical engineering, Chemical engineering

## Abstract

The influence of small changes to water hardness on the nonlinear behaviour of liquid penetration into a capillary and the resulting air pressure fluctuations during air bubble formation are examined in this paper. Experiments were undertaken in which bubbles were generated both in water having a surface tensile force of *σ* = 72.2 mN/m and in an aqueous solution of calcium carbonate having a surface tensile force of *σ* = 75.4 mN/m, each contained in a glass capillary with an internal diameter of 1 mm. It is shown that both the maximum value of liquid penetration into the capillary and bubble growth time are affected by perturbations to the water hardness. The time it takes for the bubble to depart the capillary was estimated using the following nonlinear data analysis methods: time delay (*τ*), attractor reconstructions, correlation dimension (*D*), and largest Lyapunov exponent (*λ*). All estimates demonstrate that the pressure fluctuations in the c–c aqueous solutions and extent of liquid solution penetration into the capillary during the time between subsequent bubble departures behave chaotically. Furthermore, this work demonstrates that the dynamics of bubble formation along with the bubble waiting time are very sensitive to small perturbation in the physical properties of the liquid, and this sensitivity has a significant effect on the observed chaotic behaviour.

## Introduction

The process of gas bubble formation in liquids has been investigated since the 1950s. These studies included investigations into bubble formation in bubble-beds and foam beds^1^; experiments to determine the effects of factors such as liquid surface tension, liquid viscosity, liquid density, and gas-flow rates on bubble size^2–4^; and the application of results from chaos theory to understand the dynamics of the bubble departure process^5–7^. Because the chaotic nature of the gas bubble departure process is influenced by many factors, some presently unknown, these studies continue. Moreover, understanding the dynamics of gas bubble flow in liquids is important for the chemical process and pharmaceutical industries, in which applications ranging from water aeration to various mass transfer operations rely on the careful control of such flow^8^. Also, a better understanding of the flow of methane gas bubbles in underwater environments is important for quantifying the contribution that methane release to the atmosphere has on the global balance of greenhouse gases^9,10^. In all of these applications, chaotic behaviour is observed, and water hardness is suspected to influence this chaotic behaviour. For these reasons, the focus of this paper will be to investigate the effects that small changes to water hardness have on the chaotic behaviour of bubble flow in liquid water.

From the many investigations into the chaotic nature of bubble departure^5,6,11–16^, it can be concluded that two groups of phenomena are responsible for the occurrence of chaotic bubble departures: the particular dynamics of the bubble flow in the liquid as well as the hydrodynamics of liquid flow around the capillary^5,6,11,14–18^ and the erratic changes of pressure in the gas supply system that accompany this flow^5,11,12,19–21^; these phenomena are associated with the nonlinear dynamics of the capillary filling process. These dynamics are sensitive to the physical and chemical properties of the two-phase system, notably the viscosity and surface tension, as well as to the container geometry, and have been previously investigated^21^. Nevertheless, the influence of water hardness on the nonlinear behaviour of the capillary filling process into the capillary, a topic with which the present paper is concerned, has yet to be fully investigated.

The following terminology will be used throughout this paper. The time after which liquid has filled the capillary and before the onset of bubble formation is called the *waiting* time. The time between when a vapour bubble starts to form at the tip of the capillary and when it reaches a size at which it physically detaches from the capillary tip is called *bubble growth time*.

Previous work has demonstrated the following. Firstly, the bubble growth time depends on the forces acting on the growing bubble; these forces include the drag force^22^, the buoyancy force^23^, the surface tension force^24^, a force associated with added bubble mass^25^, and a force derived from the changing momentum of the gas within the bubble^26^. Secondly, the waiting time depends on pressure changes in the gas supply and certain boundary conditions such as the gas chamber volume^11,27^, the height of the liquid over the capillary, the length of the capillary^21^ and surface tension^27^. Thirdly, an increase in chamber volume causes an increase in the bubble departure time^17^. Fourthly, a decrease of the liquid height over the capillary causes both the bubble growth time and the waiting time to decrease^17^. Fifthly, an increase in the increase in height of the liquid above the capillary caused an increase in the depth and velocity of liquid penetration into the capillary^17^. Lastly, an increase in liquid viscosity causes a corresponding increase in the bubble growth time but does not affect the bubble waiting time^17^. In all, the influence of small water hardness perturbations on liquid penetration into a nozzle and its impact on chaotic bubble departures has yet to be fully investigated. Those investigations can, for example, be helpful in achieving a better understanding of gas bubble departures in natural water reservoirs, where water hardness can significantly affect methane bubble formation in and subsequent emission from these reservoirs.

In the present paper, the liquid penetration into the glass capillary with an internal diameter of 1 mm was studied. In the experiment, bubble generation in both tap water (*σ* = 72 mN/m) and in an aqueous solution of calcium carbonate (c–c aqueous solutions, *σ* = 75 mN/m) were investigated to determine the effects of small modifications of water hardness on the bubble dynamics. The temperature of both liquids was equal to 20 °C. The depth of liquid penetration into the capillary was filmed with a high-speed camera, and pressure fluctuations in the gas supply system were measured for different air volume flow rates. Bubble departure periodicity was estimated using the following non-linear data analysis methods: time delay (*τ*), attractor reconstructions, correlation dimension (*D*) and largest Lyapunov exponent (*λ*). In “Experimental setup” section of this paper, the experimental setup and data characteristics are described. Results of the experimental data analysis are shown in “Analysis of experimental data” section. In “Numerical simulations” section, a discussion of how water hardness perturbations influence bubble departure and liquid penetration into the capillary is given. Finally in “Discussion” section, the periodicity of bubble departures is discussed.

## Experimental setup

**Figure 1:**
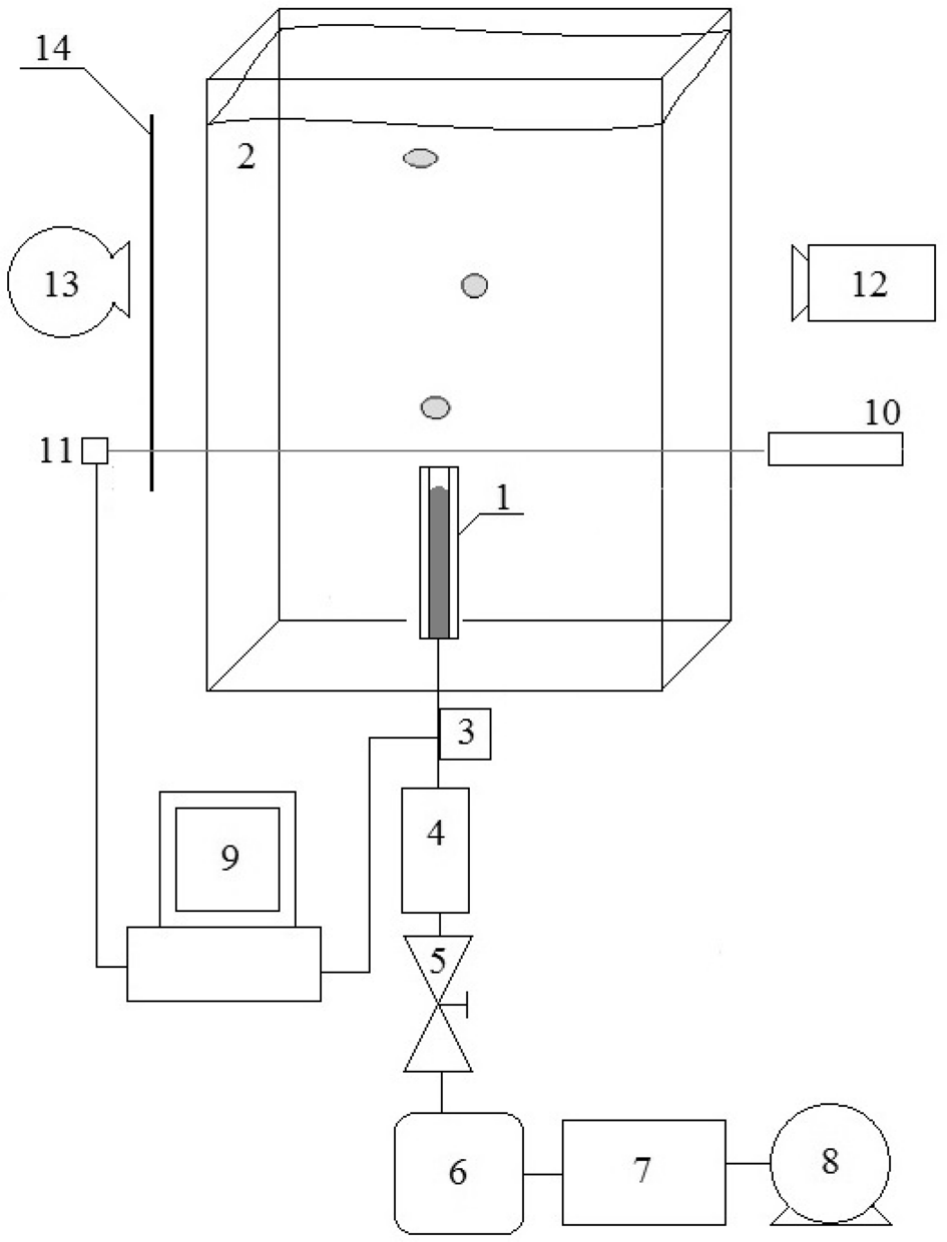
Schematic of the experimental setup. 1—glass capillary, 2—glass tank, 3—pressure sensors, 4—flowmeter, 5—air valve, 6—air tank, 7—pressure regulator, 8—air pump, 9—computer acquisition system, 10—laser, 11—phototransistor, 12—high speed camera, 13—light source, 14—screen.

**Figure 2:**
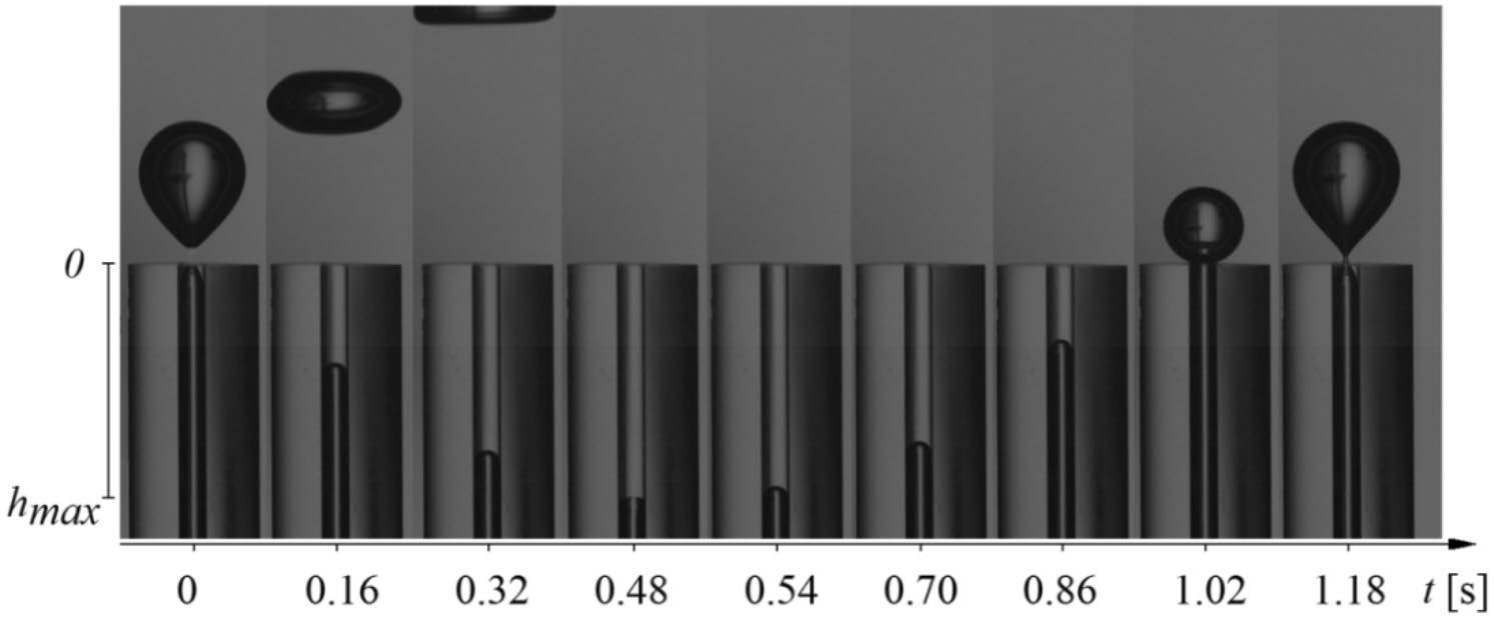
Subsequent video frames of water penetration into a capillary for an air volume flow rate of 0.014 l/min (the air is represented by dark pixels).

A schematic of the experimental setup is shown in Fig. 1, in which all components described subsequently are shown. Air bubbles were generated from a glass capillary placed in a bottom of the tank 300 × 150 × 700 mm, filled either with water or a c–c aqueous solution, as shown in Figs. 1 and 2. The surface tensile force of water was established as 72.2 mN/m and that of the c–c aqueous solution as 75.4 mN/m using an STA 1 Tensiometer, with accuracy of ± 0.1 mN/m. Values of the surface tension forces of both liquids were determined by averaging measurements made on three samples of each. The length of the capillary was equal to 75 mm, and its inner diameter was equal to 1 mm. In the experiment, the temperature of the liquid was maintained at 20 °C using the digital thermometer MAXIM DS18B20, which has an accuracy of 0.1 °C.

The air volume flow rate was adjusted using an air valve and was measured by using a BRONKHORST flowmeter. An air tank was powered by an air pump. The pressure in the air tank was set using a proportional pressure reducing valve supplied by Metalwork Regtronic. The air pressure was set as 0.3 bar, with an accuracy equal to 0.5%.

**Figure 3:**
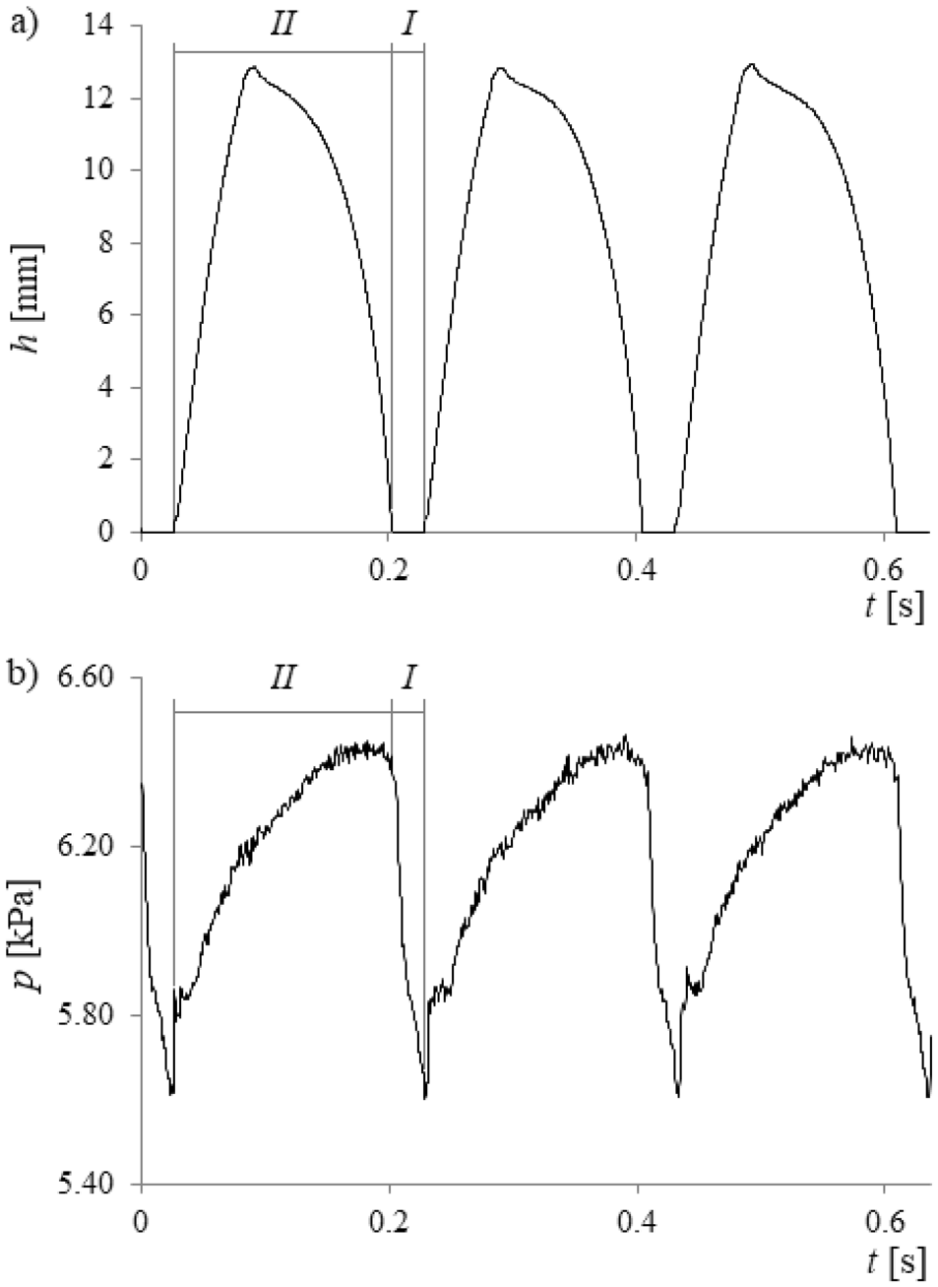
Example of recorded time series. (**a**) Liquid movement inside the glass capillary, (**b**) resulting pressure changes in the gas supply system.

During the experiment, time series of air pressure fluctuations and videos of liquid movement inside the glass capillary were recorded simultaneously. Air pressure fluctuations were measured using a silicon pressure sensor from Freescale Semiconductor MPX12DP (sensitivity was 5.5 mV/kPa). Time series of the air pressure changes were recorded by using a Data Translation DT9804 data acquisition system. The frequency of sampling was equal to 1 kHz, a maximum sampling frequency for the air pressure sensors used. The liquid movement inside the capillary was recorded with a high-speed camera—Phantom v1610. Videos were recorded with a speed of 5000 fps in the grayscale. Resolution of the video frames was 512 × 768 pixels, and the duration of each video was 15 s. In Fig. 2, a select number of frames are shown for better visualisation of the bubble departure process. A single cycle of bubble growth and liquid movement inside the capillary was recorded using 640 frames (0.118 s). Data from the high-speed camera and data acquisition station were synchronized using a laser—phototransistor system. The method of synchronization has been described in the literature^28^. Examples of the synchronized time series are shown in Fig. 3 below.

Videos were divided into frames, which were used to determine time series of the liquid movement inside the capillary. The depth of the liquid penetration into the capillary was measured by the program, which counted the number of pixels that showed the presence of water in the capillary. Pixels with brightness higher than a certain threshold indicated the presence of air in the capillary; otherwise, the pixel brightness indicated the presence of water in the capillary. Some time series representing the depth of liquid flow into the capillary and air pressure fluctuations are shown Fig. 3.

In Fig. 3, two stages of the times series are indicated using the symbols *I* and *II*. The bubble growth stage is marked using symbol *I*. During this stage, the depth of the liquid penetration is equal to 0 (Fig. 3a), and the growing bubble causes the air pressure in the gas supply system to decrease (Fig. 3b). The waiting time stage is marked using the symbol *II*. During this stage, the liquid penetrates into the capillary first, and then the liquid exits the capillary (Fig. 3a); furthermore, the air pressure decreases.

## Analysis of experimental data

**Figure 4:**
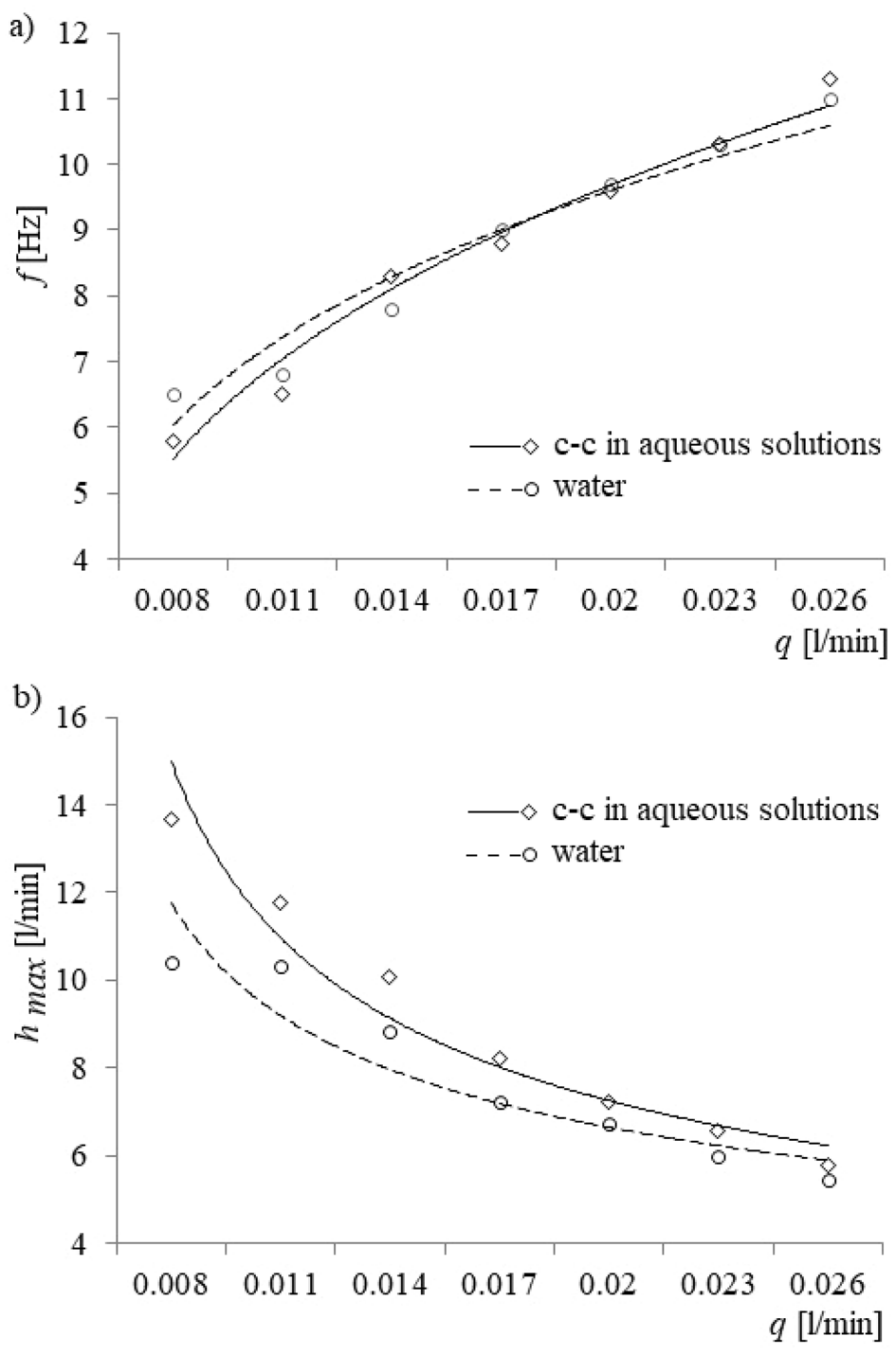
Frequencies of bubble departures and maximum depth of liquid penetration into the capillary vs. air volume flow rate. (**a**) Frequencies of bubble departures, (**b**) depth of liquid penetration into the capillary.

The frequencies of the bubble departures were estimated using the Fast Fourier Transform (FFT) method^29^. Accordingly, the original time series of pressure changes was sampled at a frequency of 1 kHz, which was the dominant frequency of the power spectrum and assumed to be the mean frequency of bubble departures. Frequencies of bubble departures vs. air volume flow rate are shown in Fig. 4a. In Fig. 4b, the maximum values of depth of liquid penetration vs air volume flow rate are shown.

The results show that the influence of surface tension on bubble departure frequency is insignificant. One notices, however, that the surface tension has a marked influence on the depth of liquid penetration into the capillary, as can be expected. The ranges of air volume flow rates, in which the liquid penetration inside the capillary occurred, varied depending on the hardness of the liquid water. When bubbles were generated in water, liquid penetration inside the capillary was observed for air volume flow rates in the range of 0.005–0.038 l/min. The largest range of air volume flow rate at which liquid movement occurs in calcium carbonate in aqueous solutions was 0.005 l/min ≤ *q* ≤ 0.044 l/min. The accuracy of the measurements was estimated by the following formula:

1 $${\sigma }_{\%}=\frac{\sigma }{{x}_{mean}}\times 100\%,$$

where $\sigma$ is the standard deviations estimated from 5 samples and *x*_*mean*_ is the mean value of all samples. The accuracy of measurements of the depth of liquid penetration as a function of air volume flow rate varied from 0.14 to 5.61% for water and from 0.41 to 3.05% for calcium carbonate in aqueous solutions. Overall, an increase of air volume flow rate caused the maximum depth of liquid penetration inside the glass capillary to decrease, a result that can be expected from the principles of fluid mechanics and discussed in “Numerical simulations” section.

**Figure 5:**
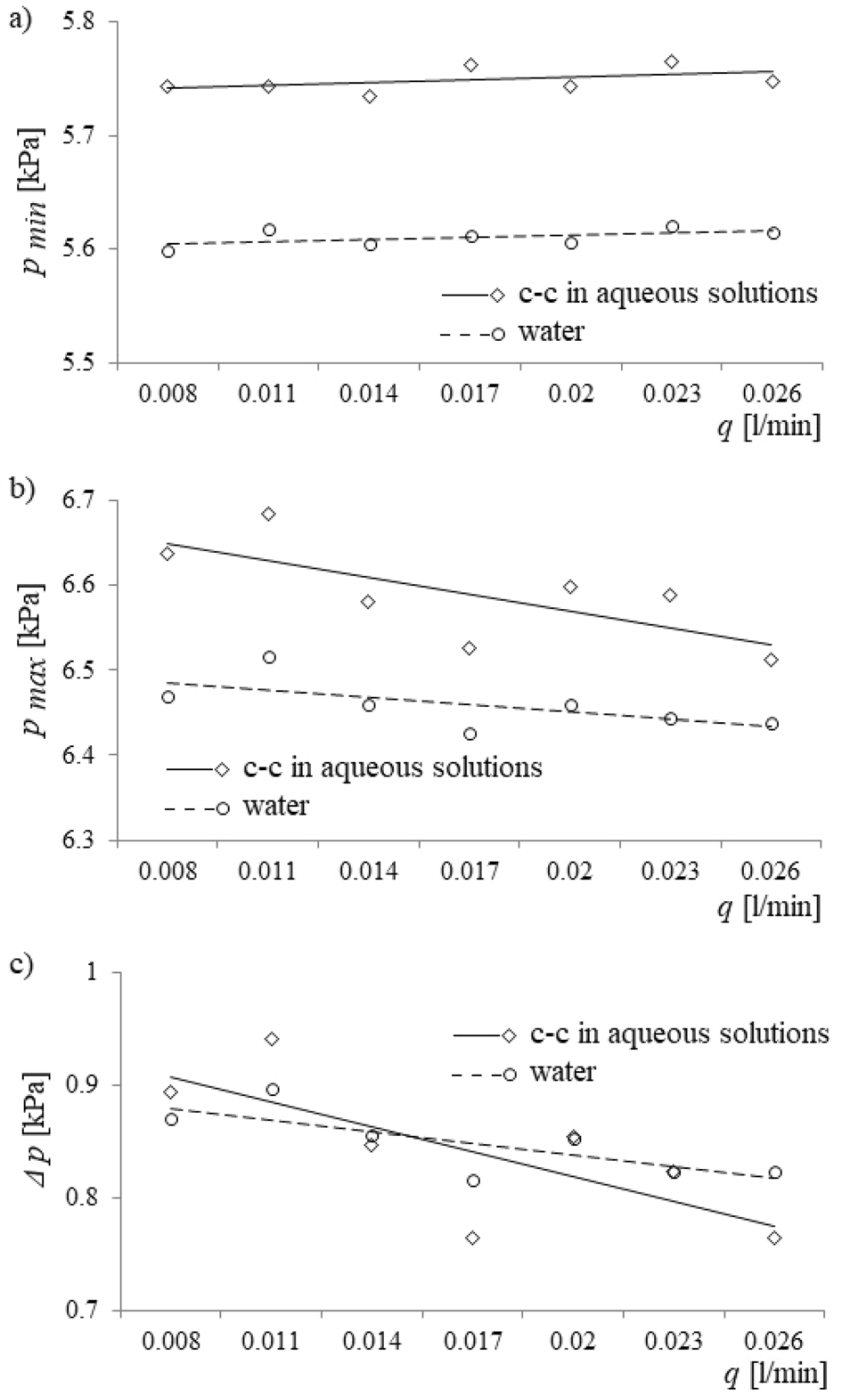
Maximum and minimum values of pressure in the gas supply system and Δ*p* vs. air volume flow rate. (**a**) Minimum pressure vs. air volume flow rate, (**b**) maximum pressure vs. air volume flow rate, (**c**) Δ*p* vs. air volume flow rate.

Maximum and minimum values of pressure in the gas supply system and Δ*p* vs. air volume flow rate are shown in Fig. 5.

At the maximum air pressure, ${p}_{max}$, bubble growth begins, and at the minimum of air pressure, ${p}_{min}$, liquid begins to enter the capillary. An increase of the air volume flow rate caused a decrease in $\Delta p= {p}_{max}-{p}_{min}$, with lower values of p_max_ and higher values of p_min_ observed and discussed in “Discussion” section. Furthermore, maximum and minimum values of pressure during bubble departure in water are lower than during bubble departure in c–c aqueous solutions. The accuracy of measurements of the values: ${p}_{max}$, ${p}_{min}$, and $\Delta p$ are presented in Table 1.

**Table 1:** Measurement accuracies of ${p}_{max}$, ${p}_{min}$, and $\Delta p$ for water and calcium carbonate in aqueous solutions.

	*p*_*max*_ (%)	*p*_*min*_ (%)	*Δp* (%)
Water	0.16–0.94	0.16–2.54	2.03–14.48
c–c in aqueous solutions	0.10–0.44	0.17–1.41	1.70–11.72

**Figure 6:**
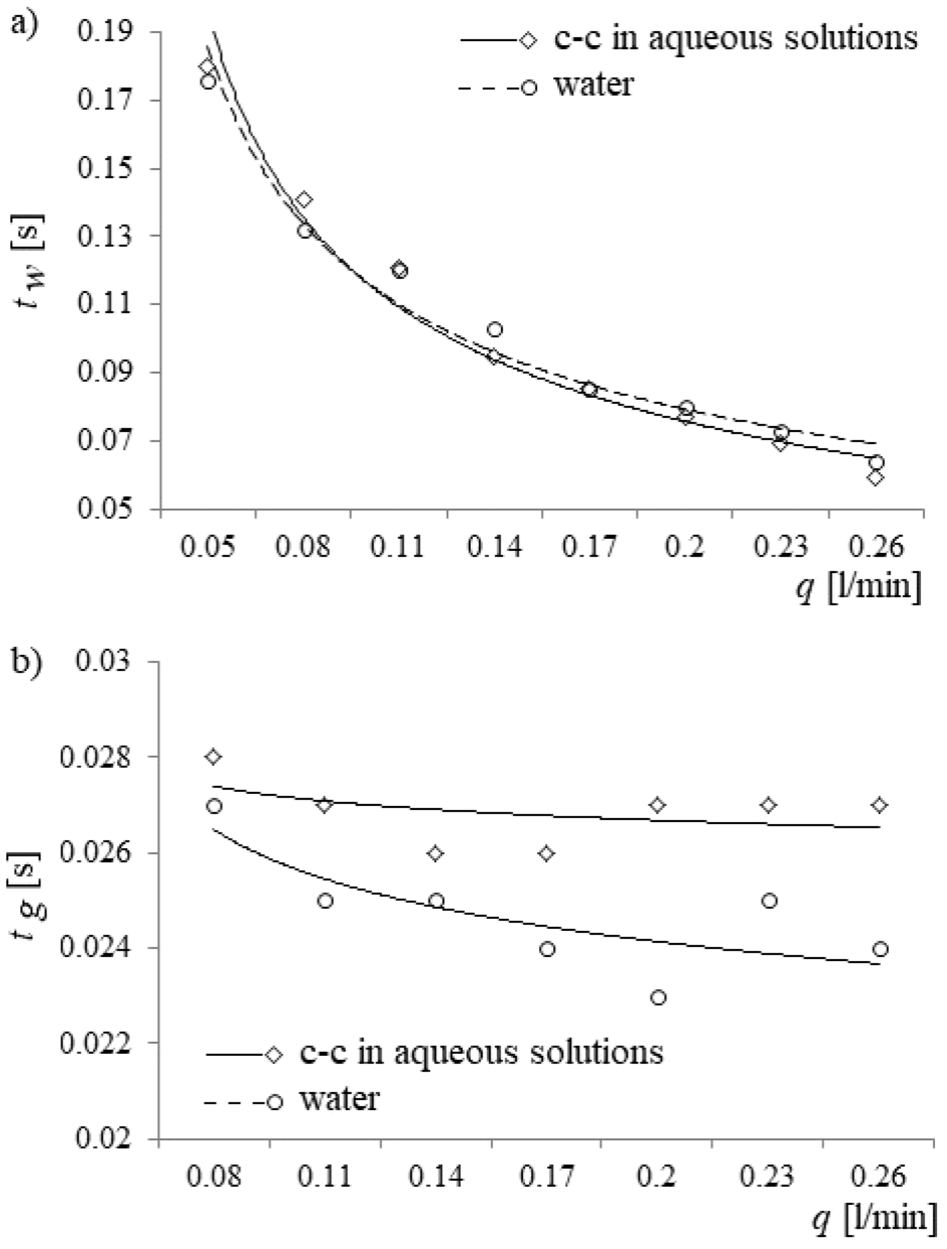
The bubble waiting time, the bubble growth time and the bubble cycle time vs. air volume flow rate. (**a**) Bubble waiting time, (**b**) bubble growth time.

In Fig. 6, the bubble waiting time and the bubble growth time vs. air volume flow rate are presented.

Waiting time and bubble growth time were estimated using the time series of liquid movement inside the capillary and compared with times estimated using time series of pressure changes in the gas supply system (examples of the time series are shown in Fig. 3). One can conclude that the presence of calcium carbonates in water changes the waiting time slightly (Fig. 6a), but significantly modifies the bubble growth time (Fig. 6b).

Measurement accuracies of the bubble waiting time varied from 0.26 to 1.54% for water and from 0.32 to 1.08% for calcium carbonate in aqueous solutions. The accuracy of measurements of the bubble growing time ranged from 0.41 to 1.75% for water and from 0.46 to 2.90% for calcium carbonate in aqueous solutions.

**Figure 7:**
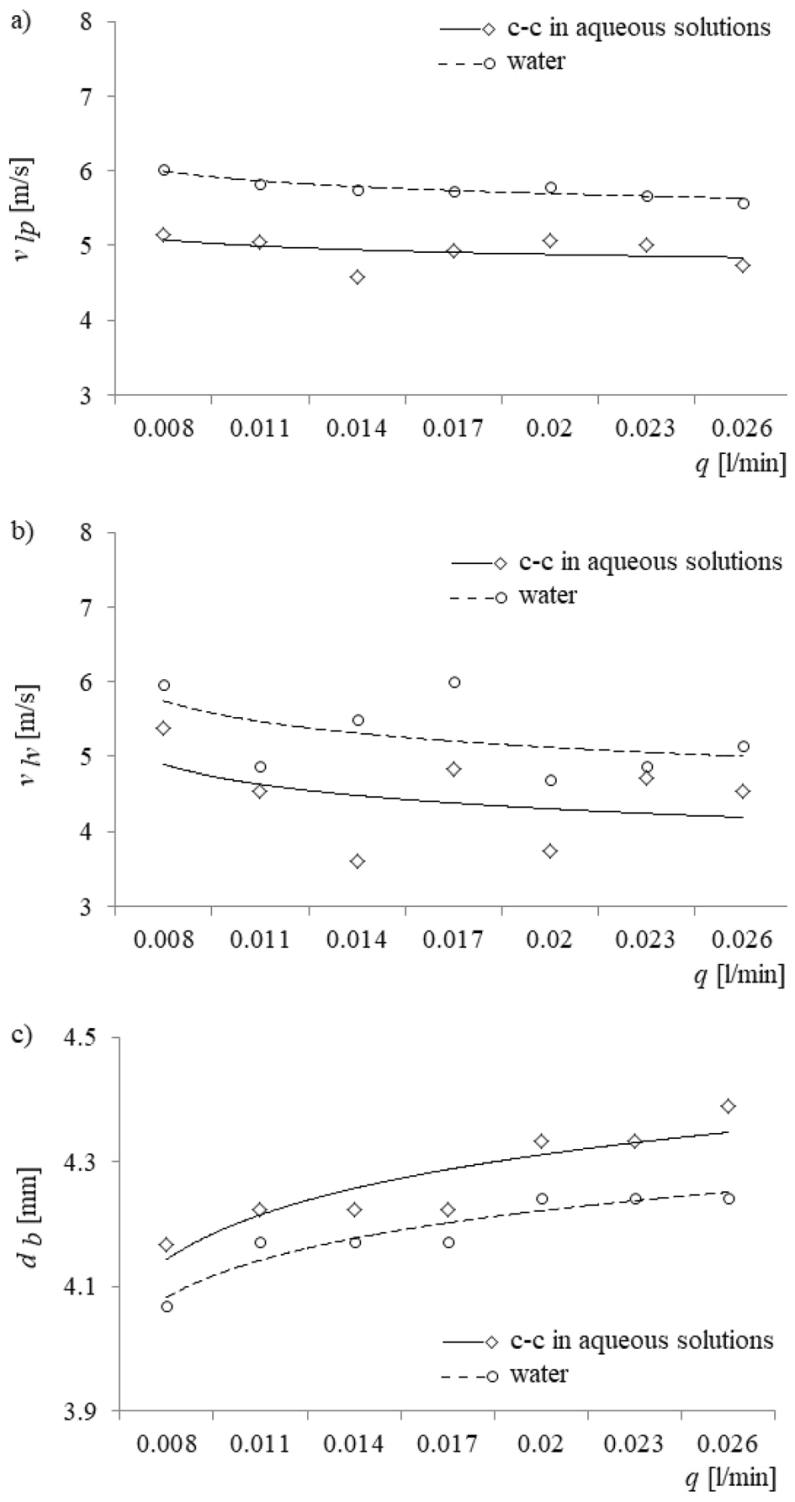
Bubble diameter growth, the exit velocity of liquid vapour from a capillary, and liquid penetration into the capillary vs. air volume flow rate. (**a**) The velocity of liquid vapour exiting the capillary, (**b**) the velocity of liquid penetration into the capillary, and (**c**) bubble diameter growth.

In Fig. 7, the velocity with which the liquid vapour exits the capillary (*v*_*lv*_), the velocity with which the liquid penetrates into the capillary (*v*_*lp*_), and the dependence of the bubble diameter (*d*_*b*_) growth vs. the air volume flow rate are shown.

Measurement accuracies of bubble diameter growth ranged from 0.38 to 0.61% for water and from 0.58 to 1.00% for calcium carbonate in aqueous solutions; those of the exit velocity of liquid vapour from a capillary varied from 0.26 to 6.50% for water and from 0.44 to 6.06% for calcium carbonate in aqueous solutions. The accuracy of measurements of the liquid penetration into the capillary varied from 0.07 to 1.35% for water and from 0.73 to 2.94% for calcium carbonate in aqueous solutions.

## Numerical simulations

**Figure 8:**
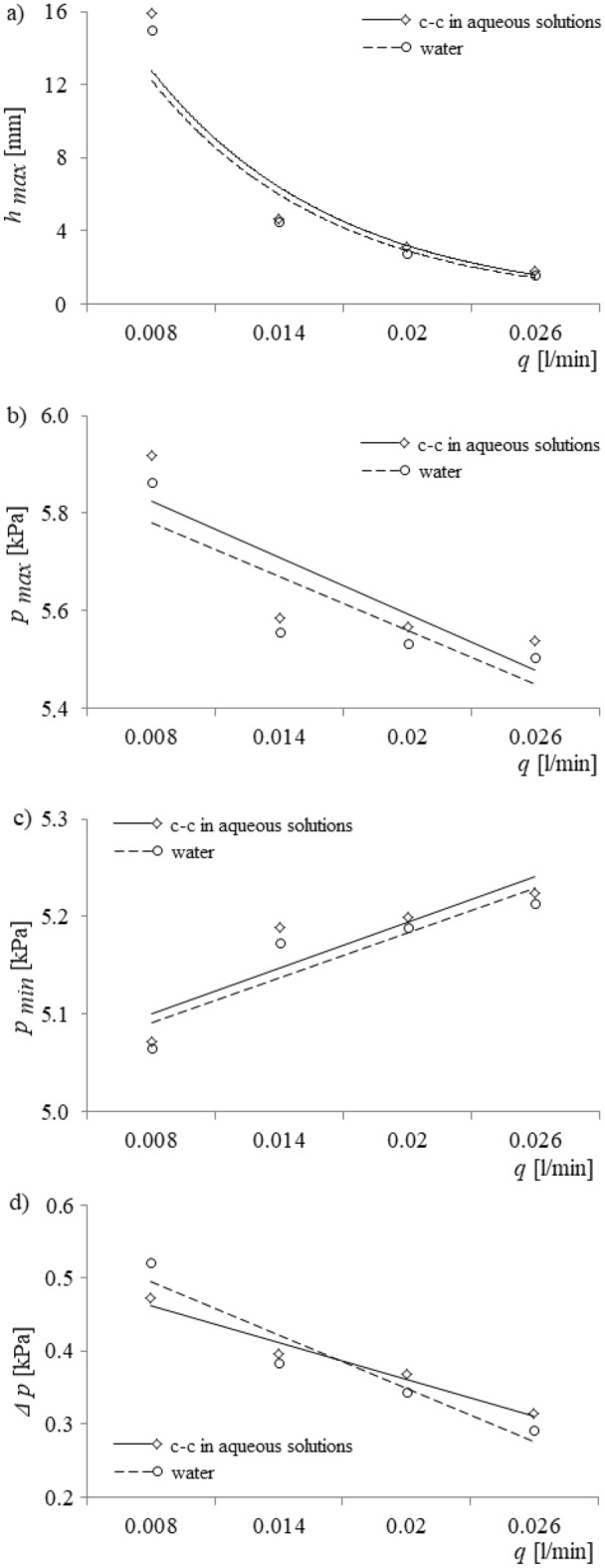
Model simulation results for the depth of liquid penetration into the capillary, maximum and minimum values of pressure in the gas supply system, and *Δp* vs. air volume flow rate. (**a**) Maximum depth of liquid penetration into the capillary, (**b**) minimum pressure vs. air volume flow rate, (**b**) maximum pressure vs. air volume flow rate, (**c**) Δ*p* vs. air volume flow rate.

In order to verify the results of the experimental investigations, results therefrom were compared to predictions from a model of bubble growth and liquid movement inside a capillary^11^. The model accounts for the forces acting on a growing bubble (the surface tension force, drag force, buoyancy force, added mass force and gas momentum), pressure changes in the capillary, that portion of the liquid mass penetrating into the capillary, and the velocity of liquid flow above the nozzle. The model is very sensitive to boundary conditions; therefore, the initial minimum value of pressure in the gas supply system was set at 5 kPa. During the model simulations, the air volume flow rate was varied between 0.008 l/min, 0.014 l/min, 0.020 l/min and 0.026 l/min. From the model simulations, time series of pressure changes in the capillary and time series of liquid penetration into the capillary were calculated. Based on these time series, the maximum depth of liquid penetration into the capillary, maximum and minimum values of pressure in the gas supply system, and Δ*p* vs. air volume flow rate were estimated. The results of these simulations are shown in Fig. 8.

Results indicated that the surface tension force significantly influenced the depth of liquid penetration into the capillary (Fig. 8a). In fact, the largest impact occurred for the low air volume flow rate, and as the air volume flow rate was increased, the effect of the surface tension force became less. Moreover, the minimum and maximum value of air pressure in the gas supply system were higher for bubble departures in c–c aqueous solutions than in water (Fig. 8b,c). Also, an increase in air volume flow rate caused the minimum values of pressure to increase and the maximum values of pressure to decrease. Last, an increase in the air volume flow rate caused a decrease in *Δp* (Fig. 8d). The results of the experimental studies were consistent with model predictions.

## Discussion

In this section, the influence of water hardness perturbations on liquid penetration into the capillary and bubble departure will be elucidated. Firstly, from Fig. 5a,b, one notices that bubble departures in a container filled with an aqueous solution of calcium carbonate are accompanied by higher maximum and higher minimum values of air pressure in the gas supply system than in a container filled with pure water. These variations in the maximum and minimum values of the air pressure are the cause of the changes observed in bubble departure and liquid penetration into the capillary. Moreover, the causes for this increase in air pressure are changes in both: (a) the forces acting on the growing bubble and (b) the liquid movement inside the capillary, as will be discussed separately in “Effects of the bubble force on the air pressure value” and “Effects of the liquid movement into the capillary on the air pressure value” sections below. Understanding the reasons for the differences between the air pressure in systems using pure water and those with aqueous solutions, as discussed in the following two subsections, allows for an understanding of the influence of water hardness on liquid penetration into the capillary and bubble departure.

### Effects of the bubble force on the air pressure value

The time at which the air bubble leaves the tip of the glass capillary corresponds to the moment at which the maximum surface tension force acting at the air–liquid interface of the bubble and described by the following equation^30^ is reached:

2 $$F_{\sigma } = 2\pi \;r_{n} \sigma ,$$

where: *σ* is the surface tension (N/m), and *r*_*n*_ is the capillary diameter (m). For the water, the maximum value of this surface tension force is approximately 2.27 mN, and for the aqueous solution of calcium carbonate, the maximum value of this surface tension force is approximately 2.37 mN; accordingly, the air pressure in the capillary will reach a higher value when the liquid is the aqueous solution than when it will for the pure water. Furthermore, because it takes longer for the larger maximum surface tension force to be reached in a calcium carbonate solution than for the smaller maximum surface tension force in pure water, the air bubble in the former solution has more time to grow; therefore, the air bubble radius grows to a greater value (Fig. 7c). The accompanying increase in air bubble volume results in a change to the buoyancy force, *F*_*B*_, acting on the bubble and to a change in the force acting on the bubble from the added mass, *F*_*AM*_; these forces are described by the following two equations^23,30^:

3 $$F_{B} = g\left( {\rho_{l} - \rho_{g} } \right) \cdot V_{b} ,$$

and

4 $$F_{AM} = - \rho_{l} \frac{d}{dt}\left[ {C_{M} V_{b} \left( {\frac{{dx_{c} }}{dt} - v_{pp} } \right)} \right],$$

where: *g* is the acceleration due to gravity (m/s^2^), *V*_*b*_ is the bubble volume, *ρ*_*l*_ is the density of the liquid (kg/m^3^), *ρ*_*g*_ is the density of gas (kg/m^3^), *C*_*M*_ = 0.5 is the added-mass coefficient for a sphere, *v*_*pp*_ is the velocity of the liquid around the bubble relative to the bubble’s expanding shell (m/s), and *x*_*c*_ is the position of the bubble centre (m). The bracketed term in Eq. (3) represents a velocity that accounts for the motion of the bubble’s centre of mass, modified by *v*_*pp*_ to account for the motion of the liquid surrounding the bubble.

The increase of bubble volume causes an increase in the buoyancy force and a correspondingly smaller increase in the oppositely directed force from the added mass; therefore, the net upward force acting on the air bubble increases, and the air bubble experiences a greater upward acceleration. The resulting increase in air bubble velocity, as given by the bracketed term in Eq. (3), is accompanied by an increase in the velocity-dependent drag force (*F*_*d*_), given by Eq. (4)^22^:

5 $$F_{d} = 0,5C_{d} \rho_{l} \pi r_{b}^{2} \left( {\frac{{dx_{c} }}{dt} - v_{pp} } \right)\left| {\frac{{dx_{c} }}{dt} - v_{pp} } \right|,$$

where *C*_*d*_ is the drag force coefficient. This larger drag force causes the last stage of bubble growth to last longer (Fig. 6b). Moreover, because the pressure in the air supply system is higher and the gas momentum (*F*_*M*_), given by Eq. (5)^24,30^, is smaller in the aqueous solution of calcium carbonate than in water, the bubble growth time is greater in the calcium carbonate solution than in water (Fig. 6b).

6 $$F_{M} = \frac{{\rho_{g} }}{{\pi r_{n}^{2} }}\left( {\frac{{dV_{b} }}{dt}} \right)^{2} .$$

### Effects of the liquid movement into the capillary on the air pressure value

Models of liquid movement inside capillaries are described in the literature^8,11,34^. In these models, two groups of forces are responsible for the process of liquid penetration into the capillary: the force *F*_1_ is related to the pressure difference that occurs in the system and the force *F*_2_ is related to the resistance of the movement of the liquid in the capillary. The small perturbations in surface tension force are responsible for changes in capillary pressure, which affect the force *F*_*1*_, described by the following equation^11,34^:

7 $${F}_{1}=-s\Delta p=-\pi {r}_{n}^{2}\left[{p}_{g}-\left({p}_{h}+{\rho }_{l}g\left(2{x}_{l}\right)+A\cdot 2\frac{\sigma }{{r}_{n}}-\frac{ \rho {v}_{pp}\left|{v}_{pp}\right|}{2}\right)\right],$$

where *x*_*l*_ is the height of the liquid penetration into the capillary (m), *s* is the cross-sectional area of the capillary (m^2^), *p*_*c*_ is the gas pressure in the plenum chamber (Pa), and *C*_*o*_ = 2 is the drag coefficient of moving water inside the capillary^21^.

On the one hand, a greater surface tension force in aqueous solutions of calcium carbonate facilitates penetration into the capillary, and the maximum depth of liquid penetration is higher than for pure water, for which the surface tension force is lower. On the other hand, a greater surface tension force hinders the removal of the liquid from the capillary. Consequently, the air velocity entering the capillary is greater for pure water than for aqueous solutions of calcium carbonate, and the effect is to lower the air pressure in the capillary when pure water is used.

## Periodicity of bubble departures

**Figure 9:**
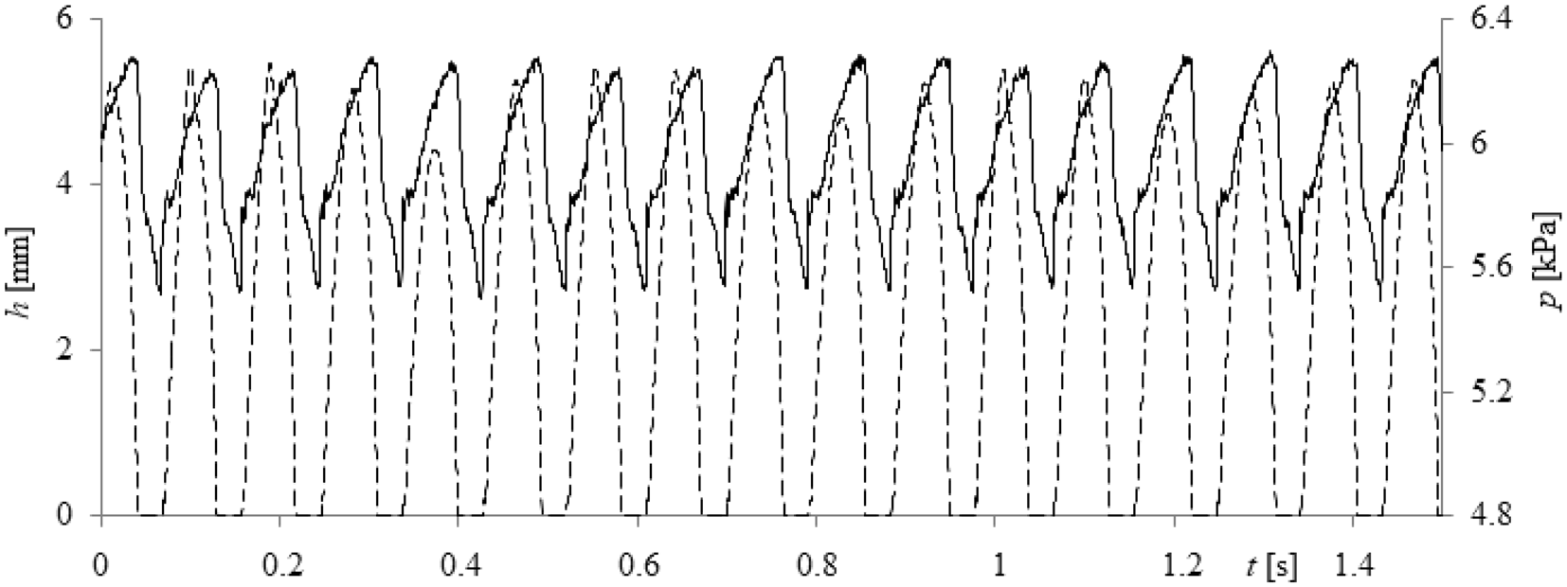
Time series of liquid penetration into the capillary and air pressure fluctuations for water, given for an air volume flow rate of *q* = 0.026 l/min. Solid lines represent pressure fluctuations in the gas, and dashed lines are used to represent the time series of liquid penetration inside the capillary.

Nonlinear methods are used to evaluate the periodicity of bubble departures. These methods include: time delay (*τ*), attractor reconstruction, correlation dimension (*D*), and largest Lyapanov exponent (*λ*). Time series data of air pressure and depth of liquid penetration into the capillary are presented in Fig. 9.

Attractor reconstruction was carried out using stroboscope coordination^22^. In this method, subsequent coordinates of attractor points are calculated based on the samples between which the distance is equal to the time delay (*τ*). The proper value of time delay (*τ*) was estimated using the following criterion^31^:

8 $${C}_{a}\left(\tau \right)\sim 0.5{C}_{a}\left(0\right),$$

with the autocorrelation function, (*C*_*a*_) of the form given by^32^:

9 $${C}_{a}\left(\tau \right)=\frac{1}{N}{\sum }_{i=0}^{n}{x}_{i}{x}_{i+\tau },$$

where *N* is the number of samples, and *x*_*i*_ is the value of *i*th sample.

The correlation dimension, *D, was* calculated using the Grassberger-Procaccia algorithm^32^.

To identify the chaotic character of the time series data, the largest Lyapunov exponent was calculated according to the following formula^33^:

10 $$L=\frac{1}{t}{\sum }_{j=1}^{m}{log}_{2}\frac{d({x}_{j+1})}{d({x}_{j})},$$

where: *m*—number of examined points, *t*—time of evolution, *d*(*x*_*j*_)—distance between points at *t* = 0, *d*(*x*_*j*_)—distance between points at *t* = *t*_*e*_.

**Figure 10:**
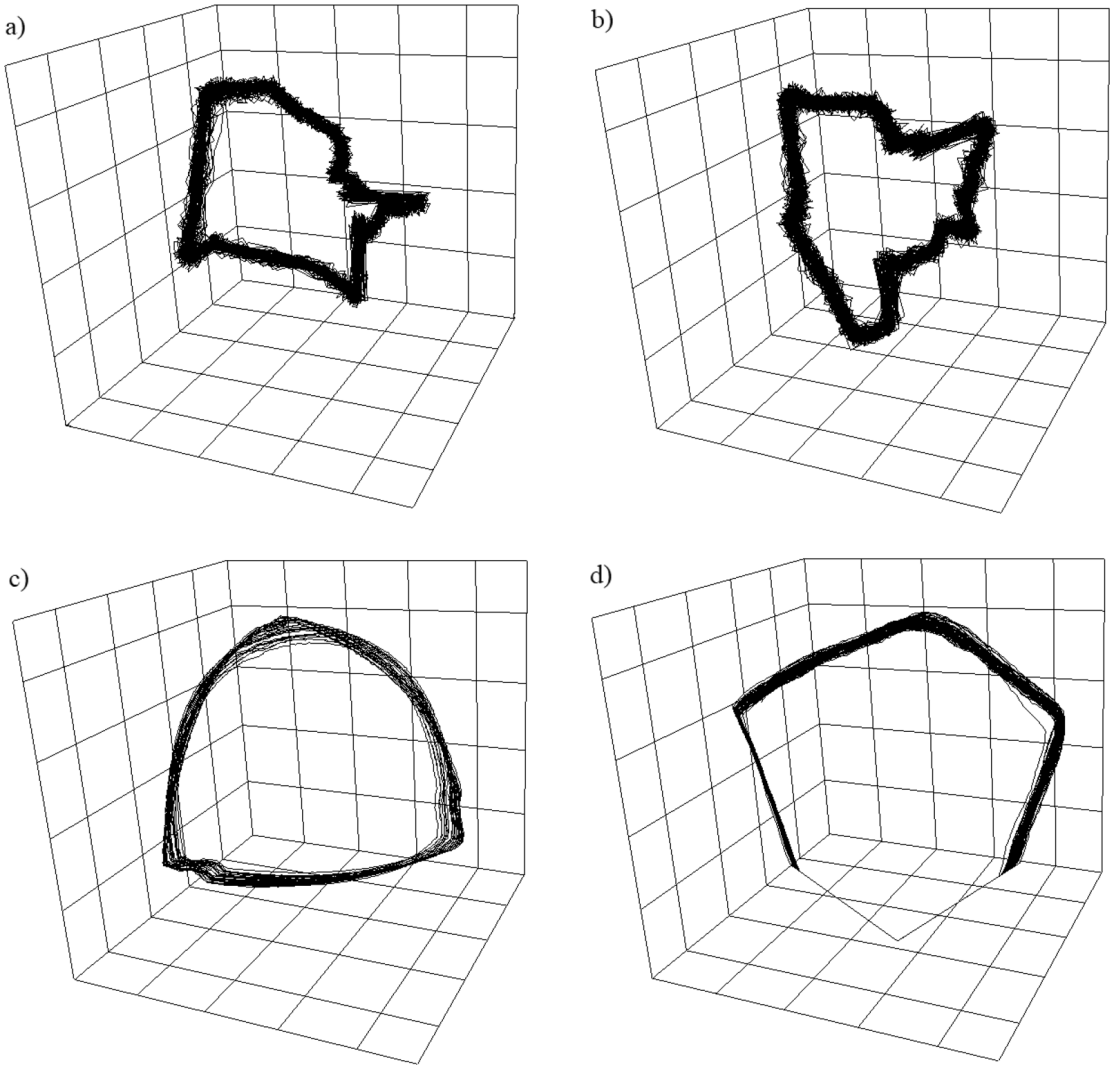
3D attractor reconstructions for water and c–c aqueous solutions at an air flow rate of q = 0.026 l/min. (**a**) Attractor reconstruction from the pressure fluctuation time series for water, (**b**) attractor reconstruction from the pressure fluctuation time series for c–c aqueous solutions, (**c**) attractor reconstruction from the time series representing the liquid penetration into the capillary for water, (**d**) attractor reconstruction from the time series representing the liquid penetration into the capillary for c–c aqueous solutions.

The 3D attractor reconstructions for both the pressure change and liquid penetration time series are presented in Fig. 10. Numerical results of these nonlinear analyses are given in Tables 2 and 3, and values of *τ* from these tables are used to reconstruct the 3D attractors for the pressure change and liquid penetration time series.

**Table 2:** Values of time delay, correlation dimension, and Lyapunov exponent calculated using the pressure fluctuations in the gas supply system at various flow rates and for both water and c–c aqueous solutions.

	*q* = 0.005 [l/min] water	*q* = 0.005 [l/min] c–c aqueous solutions	*q* = 0.026 [l/min] water	*q* = 0.026 [l/min] c–c aqueous solutions
*Τ*	25	25	14	13
*D*	3.5	3.5	3.25	3.25
*λ* [bit/s]	1.86	4.66	13.41	20.76

**Table 3:** Values of time delay, correlation dimension, and Lyapunov exponent calculated using data for the liquid penetration into the capillary at various air volume flow rates and for both water and c–c aqueous solutions.

	*q* = 0.005 [l/min] water	*q* = 0.005 [l/min] c–c aqueous solutions	*q* = 0.026 [l/min] water	*q* = 0.026 [l/min] c–c aqueous solutions
*λ*	153	147	75	71
*D*	2.5	2.5	2.5	2.75
*λ* [bit/s]	1.87	10.28	14.99	20.57

The largest bubble departure periodicity is observed for water at an air volume flow rate of 0.005 l/min. In this case, the largest Lyapunov exponent is equal to 1.86 [bit/s] for the pressure change series and 1.87 [bit/s] for the time series representing the liquid penetration into the capillary.

An increase in the air volume flow rate resulted in an increased level of chaotic behaviour for the bubbles departure. In fact, the values of the largest Lyapunov exponents for an air volume flow rate of 0.026 l/min and liquid water are: λ = 13.41 [bit/s] for the pressure fluctuations data and λ = 14.99 [bit/s] for the time series representing the liquid penetration into the capillary. For c–c aqueous solutions, pressure fluctuations and depths of liquid penetration into the capillary became more chaotic—values of λ increase.

## Conclusion

In the present paper, experiments to determine the influence of small changes to water hardness on the nonlinear behaviour of liquid penetration into a capillary and air pressure fluctuations during subsequent bubble departures were conducted, and the results compared favourably to model predictions.

The liquid penetration into the glass capillary with internal diameter of 1 mm was analysed in water (*σ* = 72 mN/m) and c–c aqueous solutions (*σ* = 75 mN/m). The conclusions of this analysis are:

- A small increase in water hardness causes an increase in the surface tension force and, therefore, an increase in the pressure at which bubbles depart.
- The surface tension force is lower for water than for aqueous solutions of calcium carbonate; therefore, air bubbles grow faster in water than in aqueous solutions of calcium carbonate.
- The increase of air volume flow rate when pure water is used causes a decrease of bubble growth time because the gas momentum increases and, therefore, the bubble departure pressure becomes lower.
- For greater air volume flow rates in systems using water, the effect of the gas momentum, which is not dependent on the water hardness, during the bubble growth stage becomes higher than the effect of the surface tension force.
- A small increase in water hardness causes the velocity of liquid movement into the glass capillary to decrease.
- An increase in the air volume flow rate causes a decrease of liquid height inside the capillary; accordingly, it can be assumed that for greater air volume flow rates, the changes of Δ*p* during a single cycle of bubble departure has a greater impact on the dynamics of liquid penetration into the capillary than the changes of surface tension force.
- For greater air volume flow rates, the differences between maximum depths of liquid penetration into the capillary decreases.

The bubble departure periodicity was estimated using the following non-linear data analysis methods: time delay (*τ*), attractor reconstructions, correlation dimension (*D*) and largest Lyapunov exponent (*λ*). For systems containing the c–c aqueous solution, pressure fluctuations and depths of liquid penetration into the capillary became more chaotic than for systems containing pure water, evidenced by increasing values of λ. Lastly, it can be concluded that:

- Small increases in water hardness cause the bubble departure process to become more sensitive to the occurrence of chaotic pressure changes and chaotic changes of liquid penetration into the capillary. Consequently, better control of gas bubble departures in applications can be achieved by careful control of a liquid’s physical parameters.
